# Efficient Synthesis of a (*Z*)-3-Methyleneisoindolin-1-one Library Using Cu(OAc)_2_•H_2_O/DBU under Microwave Irradiation

**DOI:** 10.3390/molecules18010654

**Published:** 2013-01-04

**Authors:** Li Zhang, Yongliang Zhang, Xin Wang, Jingkang Shen

**Affiliations:** 1 Instrumental Analysis Center, Shanghai Jiao Tong University, 800 Dongchuan Road, Shanghai 200240, China; 2 State Key Laboratory of Drug Research, Shanghai Institute of Materia Medica, Shanghai Institute for Biological Sciences, Chinese Academy of Sciences, 555 Zuchongzhi Road, Shanghai 201203, China

**Keywords:** (*Z*)-3-methyleneisoindolin-1-one, Cu(OAc)_2_•H_2_O/DBU, domino reaction, cross coupling, microwave irradiation

## Abstract

Microwave-promoted efficient synthesis of a (*Z*)-3-methyleneisoindolin-1-one library from 2-bromobenzamides and terminal alkynes using Cu(OAc)_2_•H_2_O/DBU is described. Various benzamide substituents, ring substitutions, including heteroaryl, aryl acetylenes and aliphatic alkynes, could be applied to afford the desired products in good to moderate yield with high stereoselectivity. It is noteworthy that DBU maybe play a dual role as not only the base, but also as a ligand for copper. The reaction is catalyzed by the complex of Cu(OAc)_2_•H_2_O and DBU without other additives.

## 1. Introduction

**Figure 1:**
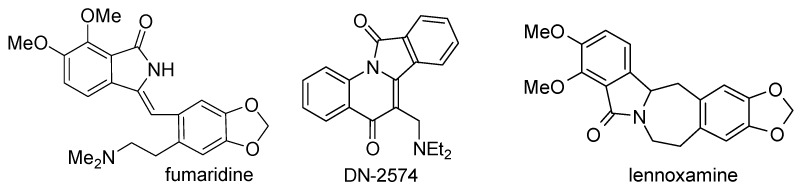
Structures of some natural products and biologically active 3-methyleneisoindolin-1-one compounds.

3-Methyleneisoindolin-1-one is an important scaffold that is present in a number of natural products and biologically active compounds (Figure 1). For example, the alkaloid fumaridine is a secophthalide-isoquinoline ene-lactam [1]. DN-2574 is an isoindoloquinoline derivative used as a cognition enhancing agent [2]. Additionally, a 3-methyleneisoindolin-1-one is a key intermediate in the total synthesis of lennoxamine [3].

There are several methods available for the synthesis of 3-methyleneisoindolin-1-ones. General methods rely on the Wittig reaction [4], nucleophilic additions of organometallic reagents to phthalimides followed by dehydration [5], Horner-Wadsworth-Emmons condensation of 3-(diphenyl-phosphinoyl)isoindolin-1-ones with aldehydes [6,7], base promoted nucleophilic additions to benzonitrile derivatives [8,9], dimethyl isoindolin-1-one-3-yl-phosphonates followed by a Horner reaction [10] or photodecarboxylative addition of carboxylates to phthalimides [11]. In recent years, efficient approaches based on palladium-mediated cyclization reactions have been reported [12,13,14,15,16,17,18,19,20]. Ma *et al.* [21] reported a CuI/L-proline-catalyzed domino reaction to form 3-methyleneisoindolin-1-ones. The desired products were formed at 85–110 °C for 24–48 h, L-proline had to be used as a ligand and K_2_CO_3_ was used as base. However, most of these methods are not suitable for parallel synthesis to obtain large numbers of (Z)-3-methyleneisoindolin-1-ones analogues for drug discovery research. As part of our ongoing drug discovery research, we required a facile route to prepare a library of (*Z)*-3-methyleneisoindolin-1-ones.

Therefore, we investigated an efficient parallel synthesis of (*Z)*-3-methyleneisoindolin-1-ones from 2-bromobenzamides and terminal alkynes in a stereoselective manner through a Cu(OAc)_2_·H_2_O/DBU catalyzed domino reaction under microwave irradiation. As microwave-assisted synthesis has become a powerful tool with the potential to improve the yields and dramatically shorten reaction times [22,23], there are several advantages to this method: (i) simple use of inexpensive complex of Cu(OAc)_2_•H_2_O with DBU, without other additives, (ii) the reaction is not sensitive to water, (iii) environmentally-friendly EtOH as the solvent, (iv) remarkably short reaction time, only 20 min being needed, (v) moderate to high yields with high stereoselectivity, only *Z*-isomers were obtained as product, and (vi), a broader substrate scope accommodating various benzamide substituents, aromatic rings including heteroaryl substitutions, aryl acetylenes and aliphatic alkynes were used. Therefore, this method are suitable to synthesize (*Z*)-3-methyleneisoindolin-1-one libraries efficiently and conveniently.

## 2. Results and Discussion

**Scheme 1:**
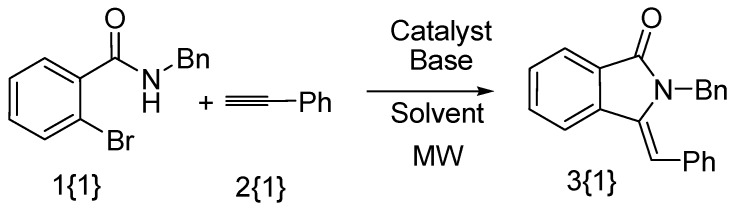
Synthesis of (*Z*)-2-benzyl-3-benzylideneisoindolin-1-one.

In order to develop an efficient methodology for the synthesis of a (*Z*)-3-methyleneisoindolin-1-one library, *N*-benzyl-2-bromobenzamide **1**{*1*} and phenylacetylene **2**{*1*} were used as representative reactants for optimization of the reaction conditions (Scheme 1). Various catalysts, bases, solvents, temperatures and reaction times were investigated (Table 1). Firstly, we compared the system of CuI and Cu(OAc)_2_•H_2_O as catalyst, the result showed that the Cu(OAc)_2_•H_2_O under microwave irradiation was better than the CuI system (Table 1, entries 1–2). Next, Cu(OAc)_2_•H_2_O was used as catalyst, and we compared different solvent effects under microwave irradiation, whereby CH_3_CN and EtOH showed similar results and better yields than DMSO, DMF or dioxane (Table 1, entries 3–7). Due to its lower toxicity and price, EtOH was chosen as the solvent for subsequent optimization. Further investigation showed that the nature of the base played an important role in the reaction process, with DBU exhibiting the best results (Table 1, entry 7). Only traces of product were detected when Cs_2_CO_3_ was used as base (Table 1, entry 10) and no product was detected in the presence of other organic or inorganic bases, such as DABCO, NEt_3_, NaOH and K_3_PO_4_ (Table 1, entries 8–9 and 11–12). DBU maybe play the dual role as not only the base, but also the ligand for copper. We also found that increasing the reaction temperature to 130 °C afforded improved yields (Table 1, entry 13). Finally, the ratio of catalyst to base was optimized. The best result was obtained when the reaction was carried out with 0.3 equiv. Cu(OAc)_2_•H_2_O and 4.0 equiv. DBU at 130 °C for 20 min under microwave irradiation (Table 1, entry 14). When the reaction time was increased to 30 min, the yield was found to decrease slightly (Table 1, entry 15).

**Scheme 2:**
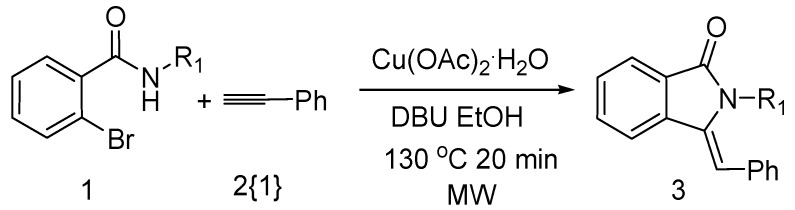
Synthesis of *N*-substituted-3-benzylideneisoindolin-1-one.

Utilizing the above optimized conditions, a library of (*Z)*-3-methyleneisoindolin-1-ones was designed and synthesized. Firstly, electronic and steric effects of various N-substituents on the 2-bromobenzamides were explored (Scheme 2). Both aromatic and aliphatic substituents were tolerated in the reaction (Table 2, entry 2–10). *N*-benzyl-substituted-2-bromobenzamides gave better yields than *N*-aryl-substituted-2-bromobenzamides (Table 2, entries 2–8). For the primary amide (R_1_ = H), the desired product was also obtained in moderate yield (Table 2, entry 1). (*Z*)-*N*-ethylheteroaryl 3-benzylideneisoindolin-1-ones could also be obtained with good yields (Table 2, entries 9–10).

**Scheme 3:**
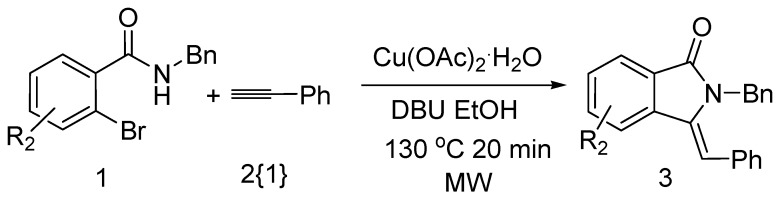
Synthesis of substituted (*Z*)-2-benzyl-3-benzylideneisoindolin-1-one.

Next, we explored the scope of substituents tolerated on the aromatic ring of the aryl bromides (Scheme 3). Good yields could be obtained regardless of the nature of the substituent (Table 3, entries 1–4). Heteroaryl bromide was also found to provide desired product in moderate yield (Table 3, entry 5).

**Scheme 4:**
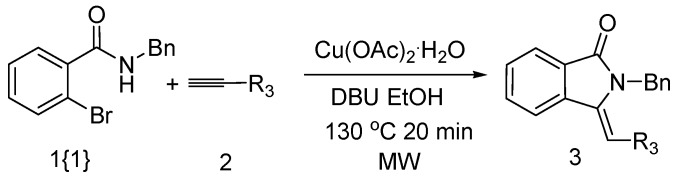
Synthesis of **3**{*17*}-**3**{*20*}.

In addition, different arylacetylenes and aliphatic alkynes were chosen to investigate the reaction scope (Scheme 4). When the triple bond was substituted with an aromatic ring, the reaction afforded better yields than that substituted with an aliphatic group. Specially, 3-methylphenyl substitution of the acetylene provided excellent result (Table 4, entry 2). 4-Fluorophenylacetylene also provided the desired product in good yield (Table 4, entry 1). Aliphatic alkynes with steric hindrance afforded the desired product in lesser yield than arylacetylenes (Table 4, entries 4).

All products were isolated in high purity and the process was highly stereoselective. All compounds were confirmed to be *Z*-isomers. The geometry of unpublished compounds was established via NOESY studies.

**Scheme 5:**
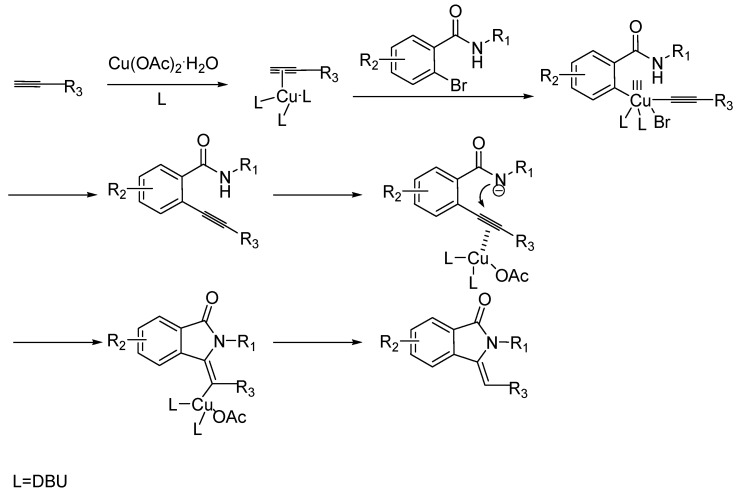
Possible Mechanism.

The possible reaction mechanism was shown in Scheme 5. The first step is likely to be the Sonogashira coupling reaction [24]. The second step involves copper coordination to the triple bond, followed by intramolecular hydroamination of the triple bond, and protodemetalation to form the desired substituted 3-methyleneisoindolin-1-one. DBU might act as ligand and base in the reaction.

## 3. Experimental

### 3.1. General 

Proton (^1^H-) and carbon (^13^C-) NMR spectra were recorded on a Varian AMX-300 spectrometer at 300 MHz and 100 MHz, respectively. Chemical shifts (δ) are reported in parts per million (ppm). Chloroform-d was used as an internal standard. Data are reported as follows: (s = singlet; d = doublet; t = triplet; q = quartet; m = multiplet; dd = doublet of doublets; br = broad). Mass spectra were carried out in EI or ESI mode. High and low resolution mass spectra were recorded on a Finnigan MAT-95 and Finnigan LCQ-Dea-XP spectrometers. Infrared (IR) spectra were obtained on a Nicolet Magan 750 FT-IR spectrometer. Analytical TLC was performed on pre-coated silica gel 60F-254 plates. Microwave-assisted synthesis was carried out in an Initiator single-mode microwave synthesizer (Biotage, Uppsala, Sweden), equipped with an internal probe that monitors reaction temperature and pressure, and maintains the desired temperature by computer control. Reactions were conducted in 5 mL sealed vials.

### 3.2. General Procedure for the Preparation of (Z)-3-Methyleneisoindolin-1-ones

Compound **2{*1*}** (0.75 mmol) was added to the mixture of Cu(OAc)_2_•H_2_O (30 mg, 0.15 mmol), DBU (293 μL, 2.00 mmol) and compound **1{*1*}** (0.50 mmol) in EtOH (1.00 mL). The reaction vessel was sealed and the reaction mixture was stirred at 130 °C for 20 min under microwave irradiation. After cooling, the mixture was diluted with EtOAc. The organic layer separated and washed with 1 N HCl, brine, dried over anhydrous Na_2_SO_4_. After evaporation under vacuum, the residue was purified by flash column chromatography (petroleum ether/ethyl acetate = 20/1–10/1) to afford the desired product. 

*(Z)-2-Benzyl-3-benzylideneisoindolin-1-one* (**3{*1*}**) [21]. ^1^H-NMR (CDCl_3_): δ 7.93 (d, *J* = 7.5 Hz, 1H), 7.74 (d, *J* = 7.8 Hz, 1H), 7.62 (t, *J* = 7.5 Hz, 1H), 7.53 (t, *J* = 7.8 Hz, 1H), 7.28–7.22 (m, 3H), 7.08–7.02 (m, 5H), 6.72 (s, 1H), 6.53 (d, *J* = 6.9 Hz, 2H), 4.94 (s, 2H); ESI-MS *m/z* 312 [M+H]^+^.

*(Z)-3-Benzylideneisoindolin-1-one* (**3{*2*}**) [21]. ^1^H-NMR (CDCl_3_): δ 8.17 (brs, 1H), 7.88 (d, *J* = 7.5 Hz, 1H), 7.80 (d, *J* = 7.8 Hz, 1H), 7.64 (t, *J* = 7.8 Hz, 1H), 7.52 (t, *J* = 7.2 Hz, 1H), 7.45–7.44 (m, 4H), 7.34–7.31 (m, 1H), 6.56 (s, 1H); ESI-MS *m/z* 222 [M+H]^+^.

*(Z)-2-(3-Fluorobenzyl)-3-benzylideneisoindolin-1-one* (**3{*3*}**). ^1^H-NMR (CDCl_3_): δ 7.93 (d, *J* = 7.8 Hz, 1H), 7.75 (d, *J* = 7.5 Hz, 1H), 7.64 (t, *J* = 7.5 Hz, 1H), 7.54 (t, *J* = 7.8 Hz, 1H), 7.29–7.22 (m, 3H), 7.06–6.97 (m, 3H), 6.81–6.77 (m, 1H), 6.74 (s, 1H), 6.33 (d, *J* = 7.5 Hz, 1H), 6.18 (d, *J* = 9.9 Hz, 1H), 4.91 (s, 2H); ^13^C-NMR (CDCl_3_) δ 168.8, 162.6 (d, *J* = 244.5 Hz), 139.4 (d, *J* = 7.1 Hz), 138.3, 134.4, 134.2, 132.3, 129.5, 129.4, 129.1, 127.9 (2C), 127.5, 123.6, 121.8, 119.5, 113.7, 113.5, 113.4, 113.1, 107.6, 44.4; IR (KBr) *ν* 3053, 1713, 1655, 1616, 1591, 1452, 1392, 1352, 1254, 1113, 955, 762, 702 cm^−1^; EI-MS *m/z* 329 (M^+^); HRMS (EI) Calcd for *m/z* C_22_H_16_NOF 329.1216, found 329.1210.

*(Z)-2-(4-Chlorobenzyl)-3-benzylideneisoindolin-1-one* (**3{*4*}**) [21]. ^1^H-NMR (CDCl_3_): δ 7.94 (d, *J* = 7.8 Hz, 1H), 7.75 (d, *J* = 7.5 Hz, 1H), 7.64 (t, *J* = 7.2 Hz, 1H), 7.55 (t, *J* = 7.5 Hz, 1H), 7.29–7.27 (m, 3H), 7.09–7.07 (m, 2H), 7.02 (d, *J* = 8.1 Hz, 2H), 6.74 (s, 1H), 6.45 (d, *J* = 8.1 Hz, 2H), 4.91 (s, 2H); ESI-MS *m/z* 346 [M+H]^+^. 

*(Z)-2-(4-methoxybenzyl)-3-benzylideneisoindolin-1-one* (**3{*5*}**) [21]. ^1^H-NMR (CDCl_3_): δ 7.91 (d, *J* = 7.5 Hz, 1H), 7.73 (d, *J* = 7.5 Hz, 1H), 7.61 (t, *J* = 7.5 Hz, 1H), 7.51 (t, *J* = 7.5 Hz, 1H), 7.29–7.28 (m, 3H), 7.13–7.12 (m, 2H), 6.71 (s, 1H), 6.58 (d, *J* = 8.4 Hz, 2H), 6.44 (d, *J* = 8.1 Hz, 2H), 4.88 (s, 2H), 3.71 (s, 3H); ESI-MS *m/z* 342 [M+H]^+^.

*(Z)-2-(Benzo[d][1,3]dioxol-5-ylmethyl)-3-benzylideneisoindolin-1-one* (**3{*6*}**). ^1^H-NMR (CDCl_3_): δ 7.91 (d, *J* = 7.2 Hz, 1H), 7.73 (d, *J* = 7.5 Hz, 1H), 7.61 (t, *J* = 7.5 Hz, 1H), 7.52 (t, *J* = 7.2 Hz, 1H), 7.31–7.29 (m, 3H), 7.15–7.13 (m, 2H), 6.73 (s, 1H), 6.48 (d, *J* = 7.8 Hz, 1H), 6.06 (s, 1H), 5.94 (d, *J* = 7.8 Hz, 1H), 5.84 (s, 2H), 4.85 (s, 2H); ^13^C NMR (CDCl_3_) δ 168.9, 147.3, 146.3, 138.5, 134.6, 134.2, 132.1, 130.7, 129.7 (2C), 129.0, 128.0, 127.9 (2C), 127.4, 123.4, 119.8, 119.4, 107.7, 107.4, 107.1, 100.7, 44.4; IR (KBr) ν 3034, 1686, 1643, 1610, 1491, 1448, 1335, 1248, 945, 771, 700 cm^−1^; EI-MS *m/z* 355 (M^+^); HRMS (EI) Calcd for *m/z* C_23_H_17_NO_3_ 355.1208, found 355.1216 .

*(Z)-3-Benzylidene-2-phenylisoindolin-1-one* (**3{*7*}**) [21]. ^1^H-NMR (CDCl_3_): δ 7.95 (d, *J* = 7.8 Hz, 1H), 7.86 (d, *J* = 8.4 Hz, 1H), 7.68 (t, *J* = 7.5 Hz, 1H), 7.55 (t, *J* = 7.8 Hz, 1H), 7.08 (m, 5H), 6.97–6.84 (m, 5H), 6.83 (s, 1H); ESI-MS *m/z* 298 [M+H]^+^.

*(Z)-3-Benzylidene-2-(4-fluorophenyl)isoindolin-1-one* (**3{*8*}**). ^1^H-NMR (CDCl_3_): δ 7.95 (d, *J* = 7.2 Hz, 1H), 7.86 (d, *J* = 7.8 Hz, 1H), 7.69 (td, *J* = 7.8, 1.2 Hz, 1H), 7.54 (td, *J* = 7.5, 0.9 Hz, 1H), 7.06–6.94 (m, 5H), 6.88–6.85 (m, 3H), 6.78–6.75 (m, 2H); ^13^C NMR (CDCl_3_) δ 168.0, 161.1 (d, *J* = 245.1 Hz), 138.4, 134.4, 133.4, 132.5, 131.8, 129.3, 129.1 (2C), 128.8, 128.7, 127.6, 127.3 (2C), 126.8, 123.9, 119.4, 115.1, 114.9, 107.6; IR (KBr) ν 3435, 3066, 1711, 1645, 1508, 1387, 1219, 1124, 760, 692 cm^−1^; EI-MS *m/z* 315 (M^+^); HRMS (EI) Calcd for *m/z* C_21_H_14_NOF 315.1059, found 315.1054.

*(Z)-3-Benzylidene-2-p-tolylisoindolin-1-one* (**3{*9*}**) [25]. ^1^H-NMR (CDCl_3_): δ 7.94 (d, *J* = 7.2 Hz, 1H), 7.85 (d, *J* = 7.8 Hz, 1H), 7.66 (t, *J* = 7.5 Hz, 1H), 7.54 (t, *J* = 7.5 Hz, 1H), 6.98–6.84 (m, 9H), 6.81 (s, 1H), 2.22 (s, 3H); ESI-MS *m/z* 312 [M+H]^+^.

*(Z)-3-Benzylidene-2-(2-(thiophen-2-yl)ethyl)isoindolin-1-one* (**3{*10*}**). ^1^H-NMR (CDCl_3_): δ 7.86 (d, *J* = 7.2 Hz, 1H), 7.77 (d, *J* = 7.8 Hz, 1H), 7.62 (td, *J* = 7.5, 1.2 Hz, 1H), 7.51 (td, *J* = 7.2, 0.6 Hz, 1H), 7.44–7.38 (m, 5H), 7.00 (dd, *J* = 4.8, 1.2 Hz, 1H), 6.83 (s, 1H), 6.79–6.76 (m, 1H), 6.32 (d, *J* = 3.3 Hz, 1H), 3.94 (td, *J* = 8.1, 2.4 Hz, 2H), 2.74 (td, *J* = 8.1, 2.4 Hz, 2H); ^13^C-NMR (CDCl_3_) δ 168.5, 139.8, 138.3, 134.7, 134.5, 132.0, 129.5 (2C), 129.0, 128.4 (2C), 128.2, 127.8, 126.6, 125.2, 123.6, 123.3, 119.3, 106.6, 42.9, 28.3; IR (KBr) ν 3390, 2939, 1701, 1664, 1614, 1471, 1444, 1346, 1097, 997, 756, 694 cm^−1^; EI-MS *m/z* 331 (M^+^); HRMS (EI) Calcd for *m/z* C_21_H_17_NOS 331.1031, found 331.1035.

*(Z)-2-(2-(1H-Pyrrol-1-yl)ethyl)-3-benzylideneisoindolin-1-one* (**3{*11*}**) ^1^H-NMR (CDCl_3_): δ 7.86 (td, *J* = 7.5, 0.9 Hz, 1H), 7.77 (d, *J* = 7.2, 0.6 Hz, 1H), 7.63 (td, *J* = 7.5, 1.2 Hz, 1H), 7.52 (td, *J* = 7.5, 0.9 Hz, 1H), 7.49–7.37 (m, 5H), 6.82 (s, 1H), 6.07 (t, *J* = 2.1 Hz, 2H), 5.97 (t, *J* = 2.1 Hz, 2H), 4.01 (td, *J* = 7.5, 1.8 Hz, 2H), 3.68 (td, *J* = 7.8, 2.4 Hz, 2H); ^13^C-NMR (CDCl_3_) δ 168.4, 138.1, 134.5, 134.4, 132.2, 129.6 (2C), 129.2, 128.5 (2C), 128.0, 127.9, 123.3, 120.6 (2C), 119.3, 108.1 (2C), 106.4, 46.6, 42.2; IR (KBr) ν 2941, 1701, 1666, 1616, 1443, 1344, 1300, 1088, 1016, 712 cm^−1^; EI-MS *m/z* 314 (M^+^); HRMS (EI) Calcd for *m/z* C_21_H_18_N_2_O 314.1419, found 314.1415.

*(Z)-2-Benzyl-3-benzylidene-5-methylisoindolin-1-one* (**3{*12*}**) ^1^H-NMR (CDCl_3_): δ 7.81 (d, *J* = 7.8 Hz, 1H), 7.54 (s, 1H), 7.34 (d, *J* = 7.5 Hz, 1H), 7.28–7.21 (m, 3H), 7.08–7.00 (m, 5H), 6.68 (s, 1H), 6.51 (d, *J* = 8.4 Hz, 2H), 4.91 (s, 2H), 2.51 (s, 3H); ^13^C-NMR (CDCl_3_) δ 169.1, 142.8, 138.8, 136.9, 134.7, 134.4, 130.1, 129.6 (2C), 127.9 (2C), 127.8 (2C), 127.3, 126.6, 126.3 (2C), 125.7, 123.3, 119.8, 107.1, 44.8, 22.1; IR (KBr) ν 3032, 1695, 1649, 1624, 1491, 1444, 1344, 1151, 968, 696 cm^−1^; EI-MS *m/z* 325 (M^+^); HRMS (EI) Calcd for *m/z* C_23_H_19_NO 325.1467, found 325.1458.

*(Z)-2-Benzyl-3-benzylidene-5-chloroisoindolin-1-one* (**3{*13*}**) [18]. ^1^H-NMR (CDCl_3_): δ 7.85 (d, *J* = 8.4 Hz, 1H), 7.71 (d, *J* = 0.9 Hz, 1H), 7.49 (dd, *J* = 8.1, 1.8 Hz, 1H), 7.29–7.25 (m, 3H), 7.06–7.04 (m, 5H), 6.69 (s, 1H), 6.50 (d, *J* = 8.1 Hz, 2H), 4.91 (s, 2H); ESI-MS *m/z* 346 [M+H]^+^.

*(Z)-2-Benzyl-3-benzylidene-6-fluoroisoindolin-1-one* (**3{*14*}**). ^1^H-NMR (CDCl_3_): δ 7.71 (dd, *J* = 8.4, 4.2 Hz, 1H), 7.59 (dd, *J* = 7.5, 2.4 Hz, 1H), 7.36–7.22 (m, 4H), 7.10–7.02 (m, 5H), 6.67 (s, 1H), 6.54–6.51 (m, 2H), 4.93 (s, 2H); ^13^C-NMR (CDCl_3_) δ 167.8, 163.4 (d, *J* = 247.8 Hz), 136.5, 134.3, 133.5, 130.0, 129.9, 129.6 (2C), 128.0 (2C), 127.5, 126.8, 126.3 (2C), 121.3 (d, *J* = 8.6 Hz), 120.0, 119.7, 110.1, 109.9, 107.8, 45.0; IR (KBr) ν 3045, 1705, 1660, 1483, 1427, 1265, 1117, 702 cm^−1^; EI-MS *m/z* 329 (M^+^); HRMS (EI) Calcd for *m/z* C_22_H_16_NOF 329.1216, found 329.1213.

*(Z)-2-Benzyl-3-benzylidene-6-methoxyisoindolin-1-one* (**3{*15*}**) [18]. ^1^H-NMR (CDCl_3_): δ 7.63 (d, *J* = 8.1 Hz, 1H), 7.39 (d, *J* = 2.1 Hz, 1H), 7.34–7.23 (m, 3H), 7.18 (dd, *J* = 8.4, 2.7 Hz, 1H), 7.09–7.05 (m, 5H), 6.60 (s, 1H), 6.54–6.52 (m, 2H), 4.92 (s, 2H), 3.93 (s, 3H); ESI-MS *m/z* 342 [M+H]^+^.

*(Z)-6-Benzyl-7-benzylidene-6,7-dihydropyrrolo[3,4-b]pyridin-5-one* (**3{*16*}**). ^1^H-NMR (CDCl_3_): δ 8.80 (dd, *J* = 4.8, 1.8 Hz, 1H), 8.20 (dd, *J* = 7.5, 1.8 Hz, 1H), 7.45 (dd, *J* = 7.5, 4.5 Hz, 1H), 7.28–7.25 (m, 4H), 7.14–7.05 (m, 5H), 6.56 (dd, *J* = 7.8, 1.8 Hz, 2H), 4.99 (s, 2H); ^13^C-NMR (CDCl_3_) δ 166.7, 156.7, 153.4, 136.4, 134.1, 133.4, 131.5, 129.5 (2C), 128.1 (2C), 127.9 (2C), 127.7, 126.9, 126.4 (2C), 123.8, 121.6, 110.0, 44.7; IR (KBr) ν 3026, 1718, 1662, 1605, 1585, 1493, 1448, 1400, 1360, 1171, 976, 781, 700 cm^−1^; EI-MS *m/z* 312 (M^+^); HRMS (EI) Calcd for *m/z* C_21_H_16_N_2_O 312.1263, found 312.1259. 

*(Z)-3-(4-Fluorobenzylidene)-2-benzylisoindolin-1-one* (**3{*17*}**) [26]. ^1^H-NMR (CDCl_3_): δ 7.93 (d, *J* = 7.2 Hz, 1H), 7.73 (d, *J* = 7.5 Hz, 1H), 7.63 (td, *J* = 6.3, 1.2 Hz, 1H), 7.56 (td, *J* = 6.3, 0.9 Hz, 1H), 7.09–6.88 (m, 7H), 6.64 (s, 1H), 6.55 (dd, *J* = 6.9, 1.8 Hz, 2H), 4.90 (s, 2H); ESI-MS *m/z* 330 [M+H]^+^.

*(Z)-3-(4-Methxybenzylidene)-2-benzylisoindolin-1-one* (**3{*18*}**). ^1^H-NMR (CDCl_3_): δ 7.93 (d, *J* = 7.2 Hz, 1H), 7.76–7.73 (m, 1H), 7.62 (td, *J* = 7.2, 1.2 Hz, 1H), 7.53 (td, *J* = 7.8, 1.5 Hz, 1H), 7.17 (t, *J* = 7.5 Hz, 1H), 7.11–7.06 (m, 4H), 6.92 (d, *J* = 7.5 Hz, 1H), 6.79 (s, 1H), 6.71 (s, 1H), 6.57 (m, 2H), 4.92 (s, 2H), 2.21 (s, 3H); ^13^C-NMR (CDCl_3_) δ 169.0, 138.5, 137.5, 136.9, 134.4, 134.2, 132.0, 130.3, 128.9, 128.1, 128.0, 127.9 (2C), 127.7, 126.6 (2C), 126.2 (2C), 123.4, 119.4, 107.7, 44.9, 21.2; IR (KBr) ν 2924, 1695, 1657, 1429, 1396, 1344, 980, 766, 698 cm^−1^; EI-MS *m/z* 325 (M^+^); HRMS (EI) Calcd for *m/z* C_23_H_19_NO 325.1467, found 325.1471.

*(Z)-3-(4-methoxybenzylidene)-2-benzylisoindolin-1-one* (**3{*19*}**) [27]. ^1^H-NMR (CDCl_3_): δ 7.92 (d, *J* = 7.6 Hz, 1H), 7.73 (d, *J* = 7.6 Hz, 1H), 7.61 (t, *J* = 7.5 Hz, 1H), 7.53 (t, *J* = 7.6 Hz, 1H), 7.09–7.06 (m, 3H), 7.01 (d, *J* = 8.8 Hz, 2H), 6.79 (d, *J* = 8.4 Hz, 2H), 6.68 (s, 1H), 6.61–6.59 (m, 2H), 4.96 (s, 2H), 3.85 (s, 3H); EI-MS *m/z* 341 (M^+^).

*(Z)-2-Benzyl-3-(cyclohexylmethylene)isoindolin-1-one* (**3{20}**). ^1^H-NMR (CDCl_3_): δ 7.89 (d, *J* = 7.5 Hz, 1H), 7.64 (d, *J* = 7.8 Hz, 1H), 7.56 (t, *J* = 8.1 Hz, 1H), 7.46 (t, *J* = 7.5 Hz, 1H), 7.32–7.19 (m, 3H), 7.12 (d, *J* = 7.2 Hz, 2H), 5.40 (d, *J* = 10.8 Hz, 1H), 5.23 (s, 2H), 2.54–2.50 (m, 1H), 1.71–1.02 (m, 10H); ^13^C-NMR (CDCl_3_) δ 168.3, 138.4, 137.6, 131.7, 131.5, 128.6 (2C), 128.2, 127.7, 127.0 (2C), 125.8 (2C), 123.3, 119.0, 115.5, 44.9, 35.7, 33.7, 32.0, 25.7, 25.6; IR (KBr) ν 2929, 2848, 1701, 1660, 1437, 1402, 1362, 1126, 968, 762, 739, 696 cm^−1^; EI-MS *m/z* 317 (M^+^); HRMS (EI) Calcd for *m/z* C_22_H_23_NO 317.1780, found 317.1773.

## 4. Conclusions 

In conclusion, we have developed a practical and convenient protocol for the preparation of (*Z)*-3-methyleneisoindolin-1-one libraries. Especially, only *Z*-isomers were obtained. This domino reaction is efficiently promoted by easily available Cu(OAc)_2_•H_2_O in the presence of DBU under microwave irradiation. Environmentally friendly EtOH was used as solvent. Various benzamide substituents, and ring substitutions including heteroaryl, aryl acetylenes and aliphatic alkynes could be applied to afford thre desired products in good to moderate yield with high stereoselectivity and high purity. Short reaction times and simple reaction conditions make this method suitable for the synthesis of (*Z*)-3-methyleneisoindolin-1-one libraries for biological and medicinal chemistry.

## Figures and Tables

**Table 1:** Optimization for the synthesis of (*Z*)-2-benzyl-3-benzylideneisoindolin-1-one ^a^.

Entry	Catalyst (equiv.)	Base (equiv.)	Solvent	Temp (°C)	Time (min)	Yield of 3{*1*} ^b^ (%)
1	CuI (0.1)	K_2_CO_3_ (2.0)	*i*-PrOH	100	20	20 ^c^
2	Cu(OAc)_2_•H_2_O (0.1)	DBU (2.0)	DMSO	100	20	30
3	Cu(OAc)_2_•H_2_O (0.1)	DBU (3.0)	DMSO	120	20	58
4	Cu(OAc)_2_•H_2_O (0.1)	DBU (3.0)	DMF	120	20	60
5	Cu(OAc)_2_•H_2_O (0.1)	DBU (3.0)	dioxane	120	20	30
6	Cu(OAc)_2_•H_2_O (0.1)	DBU (3.0)	CH_3_CN	120	20	77
7	Cu(OAc)_2_•H_2_O (0.1)	DBU (3.0)	EtOH	120	20	77
8	Cu(OAc)_2_•H_2_O (0.1)	DABCO (3.0)	EtOH	120	20	0
9	Cu(OAc)_2_•H_2_O (0.1)	NEt_3_ (3.0)	EtOH	120	20	0
10	Cu(OAc)_2_•H_2_O (0.1)	Cs_2_CO_3_ (3.0)	EtOH	120	20	trace
11	Cu(OAc)_2_•H_2_O (0.1)	NaOH (3.0)	EtOH	120	20	0
12	Cu(OAc)_2_•H_2_O (0.1)	K_3_PO_4_ (3.0)	EtOH	120	20	0
13	Cu(OAc)_2_•H_2_O (0.1)	DBU (3.0)	EtOH	130	20	83
14	Cu(OAc)_2_•H_2_O (0.3)	DBU (4.0)	EtOH	130	20	91
15	Cu(OAc)_2_•H_2_O (0.3)	DBU (4.0)	EtOH	130	30	87

^a^ Reaction conditions: **1**{*1*}(0.25 mmol), **2**{*1*}(0.375 mmol), solvent (0.50 mL); ^b^ Isolated yield; ^c^ L-proline as ligand.

**Table 2:** Synthesis of *N*-substituted-3-benzylideneisoindolin-1-one ^a^.

Entry	R_1_	Product	Yield (%) ^b^
1	H	**3{*2*}**	62
2	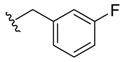	**3{*3*}**	77
3	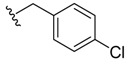	**3{*4*}**	76
4	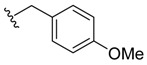	**3{*5*}**	81
5	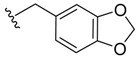	**3{*6*}**	82
6	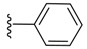	**3{*7*}**	46
7	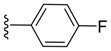	**3{*8*}**	40
8	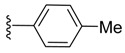	**3{*9*}**	53
9	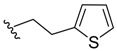	**3{*10*}**	77
10	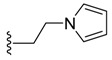	**3{*11*}**	80

^a^ Reaction conditions: **1** (0.50 mmol), **2{*1*}** (0.75 mmol), Cu(OAc)_2_•H_2_O (0.15 mmol), DBU (2.00 mmol), EtOH (1.00 mL), MW, 130 °C, 20 min; ^b^ Isolated yield.

**Table 3:** Synthesis of substituted (Z)-2-benzyl-3-benzylideneisoindolin-1-one ^a^.

Entry	Product		Yield (%) ^b^
1	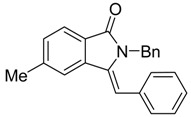	**3{*12*}**	86
2	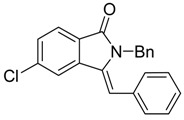	**3{*13*}**	92
3	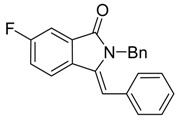	**3{*14*}**	89
4	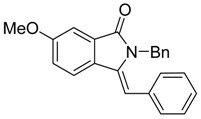	**3{*15*}**	89
5	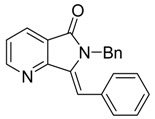	**3{*16*}**	53

^a^ Reaction conditions: **1** (0.50 mmol), **2** (0.75 mmol), Cu(OAc)_2_•H_2_O (0.15 mmol), DBU (2.00 mmol), EtOH (1.00 mL), MW, 130 °C, 20 min; ^b^ Isolated yield.

**Table 4:** Substrate Scope of alkyne in the Reaction with *N*-benzyl-2-bromobenzamide ^a^.

Entry	Alkyne	Product	Yield (%) ^b^
1	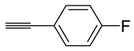	**3{*17*}**	85
2	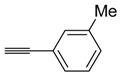	**3{*18*}**	95
3	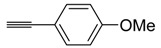	**3{*19*}**	69
4	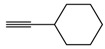	**3{*20*}**	33

^a^ Reaction conditions: **1** (0.50 mmol), **2** (0.75 mmol), Cu(OAc)_2_•H_2_O (0.15 mmol), DBU (2.00 mmol), EtOH (1.00 mL), MW, 130 °C, 20 min; ^b^ Isolated yield.

## References

[1] Rys V., Couture A., Deniau E., Grandclaudon P. (2003). First total synthesis of fumaridine. Tetrahedron.

[2] Ishihara Y., Kiyota Y., Goto G. (1990). Synthesis of Isoindolo[2,1-a]quinoline Derivatives and Their Effects on N2-Induced Hypoxia. Chem. Pharm. Bull..

[3] Fuwa H., Sasaki M. (2007). An efficient method for the synthesis of enol ethers and enecarbamates. Total syntheses of isoindolobenzazepine alkaloids, lennoxamine and chilenine. Org. Biomol. Chem..

[4] Flitsch W., Peters H. (1969). Wittig-reaktionen an imiden. Tetrahedron Lett..

[5] Bousquet T., Fleury J.F., Daich A., Netchitailo P. (2006). Oxidized isoquinolinoisoindolinone and benzazepinoisoindolinone alkaloids cores by convergent cyclisation processes: π-aromatic attack of thionium and oxonium species. Tetrahedron.

[6] Couture A., Deniau E., Grandclaudon P., Hoarau C. (2000). A New Approach to Isoindolobenzazepines. A Simple Synthesis of Lennoxamine. Tetrahedron.

[7] Moreau A., Couture A., Deniau E., Grandclaudon P. (2004). A New Route to Aristocularine Alkaloids: Total Synthesis of Aristoyagonine. J. Org. Chem..

[8] Wu M.J., Chang L.J., Wei L.M., Lin C.F. (1999). A direct anionic cyclization of 2-alkynylbenzonitrile to 3-substituted-1(2H)-isoquinolones and 3-benzylideneisoindol-2-ones initiated by methoxide addition. Tetrahedron.

[9] Lu W.D., Lin C.F., Wang C.J., Wang S.J., Wu M.J. (2002). Substituent effect on anionic cycloaromatization of 2-(2-substituted ethynyl)benzonitriles and related molecules. Tetrahedron.

[10] Reyes-Gonzalez M.A., Zamudio-Medina A., Ordonez M. (2012). Practical and high stereoselective synthesis of 3-(arylmethylene)isoindolin-1-ones from 2-formylbenzoic acid. Tetrahedron Lett..

[11] Hatoum F., Engler J., Zelmer C., Wißen J., Motti C.A., Lex J., Oelgemoller M. (2012). Photodecarboxylative addition of carboxylates to phthalimides: A concise access to biologically active 3-(alkyl and aryl)methylene-1*H*-isoindolin-1-ones. Tetrahedron Lett..

[12] Cao H., McNamee L., Alper H. (2008). Syntheses of Substituted 3-Methyleneisoindolin-1-ones By a Palladium-Catalyzed Sonogashira Coupling−Carbonylation−Hydroamination Sequence in Phosphonium Salt-Based Ionic liquids. Org. Lett..

[13] Couty S., Liegault B., Meyer C., Cossy J. (2006). Synthesis of 3-(arylmethylene)isoindolin-1-ones from ynamides by Heck–Suzuki–Miyaura domino reactions. Application to the synthesis of lennoxamine. Tetrahedron.

[14] Couty S., Liegault B., Meyer C., Cossy J. (2004). Heck–Suzuki–Miyaura Domino Reactions Involving Ynamides. An Efficient Access to 3-(Arylmethylene)isoindolinones. Org. Lett..

[15] Sun C.Y., Xu B.A. (2008). Tandem Elimination−Cyclization−Suzuki Approach: Efficient One-Pot Synthesis of Functionalized (Z)-3-(Arylmethylene)isoindolin-1-ones. J. Org. Chem..

[16] Kundu N.G., Khan M.W. (1997). An expeditious synthesis of Z-3-alkylidene isoindolinones via combined palladium catalysed and friedel-crafts reactions. Tetrahedron Lett..

[17] Kundu N.G., Khan M.W., Mukhopadhyay R. (1999). Heteroannulation through combined palladium catalysed and Friedel-Crafts reactions strategy: Synthesis of 3-alkylidene isoindolin-1-ones. Tetrahedron.

[18] Hellal M., Cuny G.D. (2011). Microwave assisted copper-free Sonogashira coupling/5-exo-digcycloisomerization domino reaction: Access to 3-(phenylmethylene)isoindolin-1-ones and related heterocycles. Tetrahedron Lett..

[19] Gabriele B., Mancuso R., Ziccarelli I., Salerno G. (2012). A new approach to isoindolinone derivatives by sequential palladium iodide-catalyzed oxidative aminocarbonylation–heterocyclization of 2-ethynylbenzamides. Tetrahedron Lett..

[20] Lee H.G., Kim K.H., Kim S.H., Kim J.N. (2013). Palladium-catalyzed oxidative arylation of trisubstituted olefin: an efficient synthesis of 3-(disubstituted)alkylidene-oxindoles. Tetrahedron Lett..

[21] Li L., Wang M., Zhang X.J., Jiang Y.W., Ma D.W. (2009). Assembly of Substituted 3-Methyleneisoindolin-1-ones via a CuI/L-Proline-Catalyzed Domino Reaction Process of 2-Bromobenzamides and Terminal Alkynes. Org. Lett..

[22] Kappe C.O., Dallinger D. (2009). Controlled microwave heating in modern organic synthesis: Highlights from the 2004–2008 literature. Mol. Divers..

[23] Jiang B., Xue L.Y., Wang X.H., Tu M.S., Liu Y.P., Tu S.J. (2012). Microwave-assisted multicomponent reaction of aryl amidines: Regiospecific synthesis of new polysubstituted thiopyrano-, and pyrano[4,3-d]pyrimidines. Tetrahedron Lett..

[24] Xie Y.X., Deng C.L., Pi S.F., Li J.H., Yin D.L. (2006). Cu(OAc)_2_/Pyrimidines-Catalyzed Cross-coupling Reactions of Aryl Iodides and Activated Aryl Bromides with Alkynes under Aerobic, Solvent-free and Palladium-free Conditions. Chin. J. Chem..

[25] Khan M.W., Kundu N.G. (1997). A Highly Regio and Stereoselective Synthesis of (Z)-3-Aryl(alkyl)idene Isoindolin-1-ones via Palladium Catalyzed Annulation of Terminal Alkynes. Synlett.

[26] Cao H., McNamee L., Howard A. (2008). Syntheses of Substituted 3-Methyleneisoindolin-1-ones By a Palladium-Catalyzed Sonogashira Coupling–Carbonylation–Hydroamination Sequence in Phosphonium Salt-Based Ionic liquids. Org. Lett..

[27] Kundu N.G., Khan M.W. (2000). Palladium-Catalysed Heteroannulation with Terminal Alkynes: A Highly Regio- and Stereoselective Synthesis of (Z)-3-Aryl(alkyl)idene Isoindolin-1-ones. Tetrahedron.

